# Factors affecting variability in vestibulo-ocular reflex gain in the Video Head Impulse Test in individuals without vestibulopathy: A systematic review of literature

**DOI:** 10.3389/fneur.2023.1125951

**Published:** 2023-03-09

**Authors:** Laurel Elise Money-Nolan, Ashley Gaal Flagge

**Affiliations:** Vestibular and Balance Lab, Department of Speech Pathology and Audiology, University of South Alabama, Mobile, AL, United States

**Keywords:** Video Head Impulse Test (vHIT), vestibular assessment, vestibulo-ocular reflex (VOR), variability in VOR gain, normative data

## Abstract

**Introduction:**

The purpose of this systematic review was to summarize and synthesize published evidence examining variations in vestibulo-ocular reflex (VOR) gain outcomes for the Video Head Impulse Test (vHIT) in healthy individuals without vestibulopathy in order to describe factors that may influence test outcomes.

**Methods:**

Computerized literature searches were performed from four search engines. The studies were selected based on relevant inclusion and exclusion criteria, and were required to examine VOR gain in healthy adults without vestibulopathy. The studies were screened using Covidence (Cochrane tool) and followed the Preferred Reporting Items for Systematic Reviews and Meta-Analyses statement standards (PRISMA-2020).

**Results:**

A total of 404 studies were initially retrieved, of which a total of 32 studies met inclusion criteria. Four major categories were identified which lead to significant variation in VOR gain outcomes: participant-based factors, tester/examiner-based factors, protocol-based factors, and equipment-based factors.

**Discussion:**

Various subcategories are identified within each of these classifications and are discussed, including recommendations for decreasing VOR gain variability in clinical practice.

## 1. Introduction

One of the primary purposes of the vestibular system is to control eye movement in response to head movement in order to maintain steady gaze on an object of interest. This process occurs *via* the vestibulo-ocular reflex (VOR), a three-neuron arc consisting of the afferent sensory vestibulocochlear nerve that is activated from the peripheral vestibular organs (semicircular canals and otolith organs), the vestibular nuclei in the brainstem, and the oculomotor nuclei (1). If functioning properly, the VOR should produce a movement of the eyes that is equal in magnitude and opposite in direction of the head impulse. The recording of these eye movements allows for a calculation of VOR gain, which is defined as the velocity of the eye movement divided by the velocity of the head movement. When the vestibular system is working optimally, the VOR gain should have a value close to 1.0, which represents that eye velocity that is equal to head velocity (2). Gain values can be as high as 1.20 (3, 4) or as low as 0.80 (5) in normal adult individuals, varying due to many pathological and non-pathological factors, with values < 0.8 typically being considered abnormal.

The Video Head Impulse Test (vHIT) is a clinical measure utilized for detecting the response of the vestibulo-ocular reflex (VOR) to angular head acceleration translations. The vHIT accomplishes this task by using video goggles to record eye movements in response to rapid, passive, unpredictable head translations delivered by a clinician. Although the vHIT cannot replace important vestibular diagnostic measures such as videonystagmography (VNG) and caloric irrigations, it has gained clinical popularity in recent years. The vHIT is useful in a vestibular test battery for determining the higher-frequency response from the semicircular canals, which are more representative of head movements encountered in daily life than simulated low-frequency head movements assessed by caloric irrigations. Equipment for the vHIT is also more cost-efficient than other vestibular tests that can evaluate such high-frequency responses from the semicircular canals, such as rotary chair testing. The vHIT has therefore become a valuable clinical assessment for these high-frequency responses in recent years, and is often chosen as the initial diagnostic test of choice in patients with vestibular disorders (2). The vHIT is useful for diagnosing vestibulopathy, which could be shown by reduced VOR gain and the presence of saccades, sometimes called reflexive or refixation saccades. These saccades are very brief eye movements to re-center the patient's eye to the target of interest after they have lagged behind in response to the initial head impulse. The vHIT is ideal for detecting these saccades, and can record both overt saccades, which could be seen with the naked eye, and covert saccades, which happen during the head motion and cannot be seen with the naked eye. In vHIT testing, the presence of saccades, along with abnormal VOR gain, can indicate vestibular dysfunction.

Although most studies evaluating normative data for the vHIT have found mean VOR gain values to be centered around 1.0 for younger adults without vestibulopathy, there is some variation in the literature. For instance, one study found that VOR gain for the horizontal canals was clustered around 1.0, especially for the youngest subjects and with lower velocity head impulses (6). For the vertical canals, however, variability in VOR gain was much greater, as shown by rapid decreases in VOR gain with increases in head impulse velocity. Another study also evaluated normative data for vHIT VOR gain (only for horizontal impulses), and found mean gain values ranging from 0.96 to 1.02 for leftward horizontal head impulses across a range of head impulse velocities (7). For rightward head impulses, gain values were higher, regardless of head velocity, with mean gain values ranging from 1.09 to 1.13. VOR gain was also minimally affected by subject age in each of these studies, but not until participants reached 70 (7) or 80 (6). In our own observations in clinical practice, we have noted many of these same variations: lower gains and increased variability for vertical canals (LARP and RALP) compared to lateral canals, a tendency toward higher gains for rightward impulses, and also variations among examiners in VOR gain and head velocity outcomes.

These clinical observations and outcomes in the literature show that there is some degree of variation in VOR gain, even for young, healthy subjects. These variations can be dependent on head impulse velocity, head impulse direction, and subject age, at minimum. There are also other considerations regarding participant characteristics, tester characteristics, protocol, and equipment which may impact VOR gain. Some of these factors are more well-known, such as goggle slippage, which can lead to inaccurate calculation of gain values. This finding has resulted in the addition of goggle tightening instructions to the test setup protocols by vHIT software manufacturers. Other considerations, such as examiner hand placement, are still being debated in the literature, but have been shown to lead to variations in VOR gain (8, 9).

Due to these factors, as well as multiple others, variations in VOR gain may be present which could impact the interpretation of results and subsequent diagnosis and treatment plan. Although normative variations on the vHIT are much smaller than normative variations on the caloric test, test developers caution that VOR gain is not an immutable and fixed number, but that it can be changed by a number of procedures (3). Since there has been evidence of VOR gain variation even in individuals without known vestibulopathy, there is potential for misdiagnosis of vestibular dysfunction if VOR gain reliability is poor or inaccurate due to a subject, tester, or protocol-related factor. Vestibular clinicians would benefit from an awareness of potential considerations that may impact VOR gain to avoid such misdiagnosis or misinterpretation. Therefore, the purpose of this systematic review is to describe, synthesize, and compare factors which may lead to variation in VOR gain in individuals without vestibulopathy as assessed by the vHIT, and to offer recommendations for decreasing variability in vHIT testing protocols. Although VOR gain is the primary focus of this study, the effects of these factors on refixation saccades will also be discussed as a secondary focus, as both reduced VOR gain and presence of saccades are typically used in vHIT diagnostic testing to diagnose vestibulopathy.

## 2. Method

### 2.1. Information sources

This systematic review followed the guidelines provided by the Preferred Reporting Items for Systematic Reviews and Meta-Analyses (10). Four academic databases were searched for relevant articles: PubMed, Scopus, CINAHL, and MedLine. Databases were initially searched in July 2021, and all obtained articles were exported, then uploaded to Covidence for abstract screening. The four databases were searched again in March 2022 to obtain any recently published relevant articles, which were also exported for screening in Covidence (2019) (11). A hand search was also completed by examining the reference lists of the articles obtained from the initial database searches.

### 2.2. Eligibility criteria

To decide whether studies should be included in this review, all abstracts obtained from database searches were reviewed by one author and compared against inclusion criteria. To be included in the review, studies were required to meet the following criteria: an original research study, utilizing the Video Head Impulse Test (vHIT) with a goggle-mounted camera system, and focusing on the vestibulo-ocular reflex (VOR) gain as the primary outcome measure in adults. While the authors recognize the utility of remote camera systems, especially in the testing of young pediatric patients (12), only goggle-mounted camera systems were included in this review, as these systems have been validated with scleral search coil measurements (13) and are currently more commonplace for use in adult populations in the U.S. As this review aimed to assess variation in vHIT VOR gain in individuals without vestibulopathy, all accepted studies included and reported results of VOR gain on healthy, asymptomatic subjects in at least one group. Studies were excluded if text was unavailable in the English language, unavailable in full text, or if a variation of vHIT other than the standard version (such as the suppression head impulse test) was used. Studies examining only pathological conditions, or examining VOR gain exclusively in pediatric populations were also excluded. Duplicate studies were automatically excluded by the Covidence (2019) (11) software used to organize and screen articles.

### 2.3. Search strategy

The four databases were searched using these relevant key terms: “Video Head Impulse Test” OR “vHIT” AND “vestibuloocular reflex” OR “vestibulo-ocular reflex” OR “VOR” AND “Normative” OR “Healthy” OR “Normal” OR “Typical” OR “Non-pathological.” All search results obtained from each database were then imported into Covidence (2019) (11) for screening of titles and abstracts by one reviewer. Articles which were screened and found to meet inclusion and exclusion criteria were obtained in full-text, if possible.

## 3. Results

### 3.1. Overall search results

**Figure 1:**
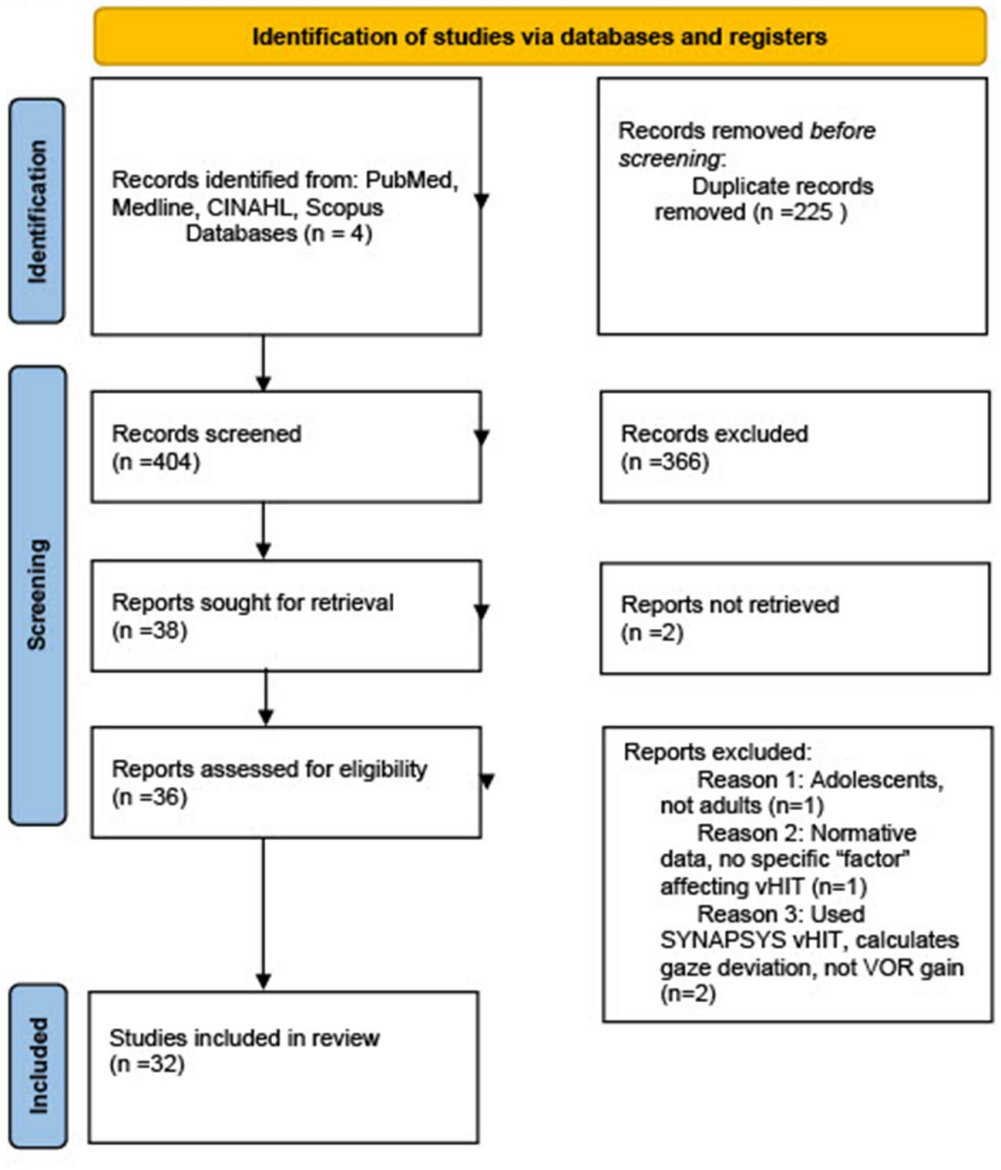
PRISMA flow diagram of studies included in systematic review. From Page et al. (10).

The initial database search yielded 404 original research articles (Figure 1). After abstracts of each article were compared to inclusion and exclusion criteria, irrelevant articles were removed, and a total of 32 original articles were attempted to be obtained in full text for thorough review. A hand search was conducted of references in the relevant articles to obtain any additional pertinent articles that were missed in the original database search, yielding an additional six articles, for a total of 38 articles for consideration to be included in this systematic review. Two of these articles were unable to be obtained in English, and two articles which used the SYNAPSYS vHIT software which uses a remote camera rather than goggles to record head and eye movements were excluded. Additionally, one article examined only adolescents rather than adults, and another listed normative values without giving any experimental data examining factors affecting VOR gain, for a total of six further articles excluded after the full-text search and review process. Therefore, a total of 32 original research articles were included in this systematic review.

The 32 articles were each reviewed independently by two researchers, who read each article and synthesized themes for each study. Detailed themes associated with variation in vHIT VOR gain were compared across studies, and classified into four major categories. The results showed that vHIT VOR gain in individuals without vestibulopathy can vary due to participant characteristics, examiner factors, vHIT test protocol, and equipment differences across manufacturers. Within each of these four major themes, multiple subcategories were noted (Table 1), with the theme of “test protocol” contributing to the greatest number of articles (*n* = 15). It should be noted that several studies examined more than one independent variable, and therefore potentially could have fallen into multiple categories (e.g., a study examined the effects of both age and hand placement on VOR gain). For simplicity, the primary factor investigated in each study, as noted in the title, abstract, and discussion, was utilized to categorize the article. However, additional relevant findings of the study are listed in Table 2. Eleven of the 32 articles presented in this study also showed significant findings related to saccades, which will be presented in the discussion, as the results will focus on VOR gain as the primary objective of this review.

**Table 1:** Participant demographics and protocol information for included studies.

		**Participant demographics**	**Distance from visual target**	**Number of impulses/canal**	**Number of examiners**	**Head movement velocity (deg/s)**	**Canals tested**
**Participant characteristics (*****n*** = **12)**							
Age	Abakay et al. (14)	*n* = 129 healthy subjects (12–88 yrs, M = 44.30, grouped by decade)	1 m	No info	1	No info	Laterals, RALP, LARP
	Kim and Kim (15)	*n =* 835 for lateral canals *n =* 434 of same subjects for vertical canals (ages 10–89, grouped by decade) ^*^All had previous hx of dizziness, but none within at least the last month; cVEMP & caloric results all WNL	1 m	15–20	1	150–250 (lateral) OR 100–200 (vertical)	Laterals, RALP, LARP
	Matiño-Soler et al. (7)	*n =* 212 healthy subjects (5–95 yrs, grouped by decade)	1 m	At least 20	1	70–90 100–120 140–160 180–200	Laterals
	McGarvie et al. (6)	*n =* 91 healthy subjects (10–89, grouped by decade).	1–1.8 m	At least 10	1	< 120; 120–180; >180 (lateral) OR < 110; 110–140; >140 (vertical)	Laterals, RALP, LARP
	Mossman et al. (16)	*n =* 63 healthy subjects (20–80 yrs, grouped by decade).	1.5 m	6–10	1	150–300	Laterals
	Pogson et al. (17)	*n =* 80 healthy subjects (16–84 yrs, M = 47, grouped by decade).	1.5 m	At least 20	1	100–300	Laterals, RALP, LARP
	Treviño-González et al. (18)	*n =* 132 normal subjects (21–79 yrs, 63 females, M = 48.44, 69 males, M = 46.43, grouped by decade).	1 m	7–15	1	150–250 m/s	Laterals
	Yang et al. (19)	*n =* 50 normal subjects (20–69, grouped by decade).	1 m	At least 10	2	150–200	Laterals
Test/Retest reliability	Bansal and Sinha (20)	*n =* 25 normal subjects (17–25 yrs, M = 22)	1 m	At least 20	1	100–200 (not reported in text, but shown in figures)	Laterals, RALP, LARP
	Singh et al. (21)	*n* = 20 healthy subjects (18–30 yrs, M = 22.2) *n =* 20 (21–80 yrs, M = 45) patients w/ vertigo	1 m	20	1	100–250 for lateral, 50–250 for vertical	Laterals, RALP, LARP
Visual acuity	van Dooren et al. (22)	*n =* 79, healthy subjects (18–80 yrs, M = 54, grouped by corrective lenses—control, spectacles, contacts)	2 m	At least 10	1	>150	Laterals
Mental state/anxiety	Naranjo et al. (23)	*n =* 25 young, healthy subjects (M = 27.8 yrs)	3.1 m	20	No info	100–200	Laterals, RALP, LARP
**Tester characteristics (*n* = 2)**							
Examiner reliability	Mutlu et al. (24)	*n =* 21 healthy subjects (age >17 yrs, M = 26.04)	5 ft	At least 12–20	4	150–300	Laterals, RALP, LARP
	Abrahamsen et al. (25)	*n =* 120 healthy subjects (18–65 yrs, M = 43.5)	1.5 m (SystemA) 1 m (System B)	15	2	Discussed, but not specifically stated as part of protocol	Laterals, RALP, LARP
**Protocol (*****n** =* **15)**							
Hand placement	Fu et al. (8)	*n =* 86 healthy subjects (19–73 yrs, M = 42.5) *n =* 67 individuals with unilateral vestibular neuritis (28–77 yrs, M = 46.71)	1.2 m	>20	1	100–200	Laterals
	Patterson et al. (9)	*n =* 20 healthy young subjects (20–39 yrs, M = 25.2) *n =* 20 healthy older subjects (51–88 yrs, M = 68)	1 m	At least 20	3	150–200	Laterals
Goggle slippage	Suh et al. (26)	*n =* 8 healthy subjects (26–33 yrs, M = 28)	1.5 m	At least 10–20	1	150–300	Laterals
Target distance/size	Castro et al. (27)	Experiment 1: *n =* 18 healthy subjects (M = 27.2 yrs) Experiment 2: *n =* 10 of the same subjects (M = 27.9 yrs).	150, 40, 30, 20, and 10 cm	15–18 valid impulses	No info	50–300	Laterals
	Jay et al. (28)	*n =* 48 healthy subjects (18–77 yrs, grouped by decade).	1.5 m	At least 15	1	150–200	Laterals
	Judge et al. (29)	*n =* 38 healthy control subjects (22–63 yrs, M-37.3, grouped by visual acuity) *n =* 8 individuals with vestibulopathy (31–65 yrs, M = 45.9)	0.6, 1.2, and 2.4 m	No info	No info	No info	No info, maybe lateral?
Head/eye position	Maxwell et al. (30)	*n =* 22 healthy subjects (M = 25.6 yrs)	1.5 m	At least 15	2	>150	Laterals
	McGarvie et al. (31)	*n =* 10 healthy, active community-dwelling subjects	1.2 m	20	1	150–200	LARP
	Patterson et al. (32)	*n =* 24 healthy control subjects (23–42 yrs, M = 32) *n =* 4 individuals with BVL (21–40, M = 32)	1.2 m	10–20	1	>100	LARP
	Seo et al. (33)	*n =* 20 healthy control subjects (24–38 yrs, M = 28.4) *n =* 18 subjects with UVL (27–64 yrs, M = 44.4)	1 m	At least 20	No info	150–200	Laterals
Thrust direction	Park et al. (34)	*n =* 24 healthy subjects (26–39 yrs, M = 30)	1 m	At least 10	1	227–245 (reported means)	Laterals
	ElSherif (35)	*n =* 20 healthy subjects (24–60 yrs, M = 37.3)	1 m	At least 2 w/ no artifacts	1	100–250	Laterals
	Nyström et al. (36)	*n =* 21 healthy subjects (17–44 yrs)	1 m	20	No info	152–159 (reported means)	Laterals
Camera placement	Strupp et al. (37)	*n =* 100 healthy subjects (19–95 yrs, M = 46.8)	180 cm	At least 20	1	No info	Laterals
Predictability of thrust	Yilmaz et al. (38)	*n =* 19 healthy subjects (20–23 yrs, M = 21.84)	No info	No info	No info	No info	Laterals
**Equipment differences (*****n** =* **3)**							
Software	Lee et al. (39)	*n =* 25 healthy subjects (M = 31)	1 m	At least 10	1	200–250	Laterals
Calculation	Janky et al. (40)	*n =* 61 healthy control subjects (20–78 yrs, M = 49, grouped by decade) *n =* 11 individuals with UVL (*n =* 8) or BVL (*n =* 3) (32–79 yrs, M = 52.3)	1 m	EyeSeeCam: At least 20 Impulse: At least 20 VisualEyes: At least 10	3 (each participant tested by 1 examiner)	100–250	Laterals
	Jacobsen et al. (41)	*n =* 60 healthy subjects (18–65 yrs, M = 42.6)	1.5 m	At least 10	2 (both tested all subjects)	150–300	Laterals

hx, history; yrs, years; BVL, bilateral vestibular loss; UVL, unilateral vestibular loss.

**Table 2:** Secondary factors examined for included studies.

**Primary factor studied**	**Study**	**Secondary factor(s) affecting VOR gain**
**Participant characteristics (*****n** =* **12)**		
Age	Abakay et al. (14)	**Sex:** Not significant **Vertical vs. horizontal canal gain:** No significant differences
	Kim and Kim (15)	**Impulse direction:** VOR gains significantly higher for right lateral than for left **Vertical vs. horizontal impulses:** Gradual decline in horizontal and posterior VOR gain w/ age; pts w/ age over 70 had significantly decreased horizontal VOR gain; no significant age differences for anterior canal **Refixation saccades:** No results reported
	McGarvie et al. (6)	**Impulse direction:** VOR gains significantly higher for right lateral than left lateral, and for right anterior than left anterior **Vertical vs. horizontal impulses:** VOR gain more variable for vertical **Impulse velocity:** Small decreases in VOR gain with increases in velocity for horizontal canals; larger decreases in VOR gain with increased velocity for vertical canals
	Matiño-Soler et al. (7)	**Head impulse velocity:** For participants >70, head impulse velocity was significantly lower; for all (both rightward and leftward) head impulses, VOR gain decreased with increases in velocity **Sex:** not significant **Reflexive saccades (RSs):** Number of subjects with RS and number of head impulses with RS significantly increased with age >71 years; mean VOR gain significantly lower in subjects with RS than without; fewer RS after rightward impulses vs. leftward (not statistically significant) **Impulse direction:** VOR gains significantly higher for right lateral than left, regardless of age or impulse velocity; gain asymmetry between left and right impulses increased significantly with age
	Mossman et al. (16)	**Time of instantaneous VOR gain analysis, 60 vs. 80 ms:** Gain statistically significantly lower at 60 ms, but not clinically significant; decline in HVOR velocity gain with increased age for both 60 and 80 ms; authors suggest 60ms is most accurate in pts w/ covert saccades **Impulse direction:** No significant differences between right and left lateral **Target distance:** VOR gain increased as target distance decreased, especially with distances < 0.70 m
	Pogson et al. (17)	**Refixation saccades:** Saccades present in all ages; increased in frequency, amplitude, and peak velocity for older subjects; anterior canal had least frequent saccades; largest amplitude saccades in posterior canal **Gain calculation methods:** Lower gain calculated in lateral canals using narrow detection window for calculation **Impulse direction:** VOR gains significantly higher for right lateral, right anterior, and left posterior canals than counterparts **Impulse velocity:** Gain decreased with increases in head velocity for all canals
	Treviño-Gonzaáles et al. (18)	**Impulse direction:** VOR gains significantly higher for right lateral than left **Gender:** Mean gain for males significantly higher with instantaneous VOR gain calculation (at 80 ms only)
	Yang et al. (19)	**Covert & overt saccades:** No statistically significant differences between age groups in presence or amplitude of saccades; overt saccades most common, found in 16.8% of head impulses **Impulse direction:** VOR gains significantly higher for right lateral than left **Gain asymmetry:** No significant differences between ages
Test/retest reliability	Bansal and Sinha (20)	**Impulse direction:** VOR gains significantly higher for right lateral than left lateral, and for right anterior than left anterior in both session 1 and session 2. VOR gains significantly higher for left anterior than right posterior in session 2 only **Horizontal vs. vertical canals VOR gain:** Gain slightly higher for horizontal vs. vertical (except left anterior) in all trials
	Singh et al. (21)	**Presence of refixation saccades & their test-retest reliability:** Reliability of RS moderate to excellent for lateral SCCS; poor to moderate for vertical SCCs
Visual acuity	van Dooren et al. (22)	**Impulse direction:** No significant difference in VOR gains for rightward vs. leftward impulses **Monocular vs. binocular recording:** No significant differences in VOR gain
Mental state/Anxiety	Naranjo et al. (23)	**Head impulse velocity:** No significant differences in velocity for high vs. low postural threat conditions **Electrodermal activity (EDA), fear, anxiety, and percieved confidence:** Significantly increased EDA, fear, and anxiety, and significantly decreased confidence for high postural threat condition vs. low threat condition
**Tester characteristics (*****n** =* **2)**		
Examiner reliability	Mutlu et al. (24)	**Gain calculation method:** Significant differences between examiners in lateral and vertical canal VOR gain for instantaneous gain calculation at 40, 60, and 80 ms; Less significant differences noted between examiners using velocity regression calculation- values similar between examiners for right lateral, right posterior, and left anterior canals. **Horizontal vs. vertical impulses:** No significant differences **Impulse direction:** No significant differences for lateral or vertical canal pairs
	Abrahamsen et al. (25)	**Test time:** Testing all six SCCs took less time with the EyeSeeCam than with the ICS Impulse; test time took longer for medical student than experienced doctor **Number of head impulses accepted by software:** Higher mean number of accepted impulses with ICS Impulse than EyeSeeCam **Impulse direction:** VOR gains significantly higher for right lateral than left, with both systems analyzed
**Protocol (*****n** =* **15)**		
Hand placement	Fu et al. (8)	**Head impulse velocity:** No significant differences in mean velocity when using head hand placement vs. jaw hand placement **Presence of “overhigh” VOR gains:** Significantly more overhigh VOR gains for head placement than jaw placement **Impulse direction:** VOR gains higher for right lateral than left (statistical significance not reported)
	Patterson et al. (9)	**Intra-rater reliability:** Acceptable reliability criterion for both gain and velocity for all three examiners in a single session **Inter-rater reliability:** In an analysis where right and left impulses were combined across hand placements, excellent reliability was found for gain with head-hand placement, and fair reliability for gain using chin-hand placement, for all ages. Fair reliability was found for chin placement head-impulse velocity, and poor reliability for head placement velocity, for all ages; In an analysis where left and right impulses were analyzed separately, excellent inter-rater reliability was found for average VOR gain using head placement with impulses in both directions. Good to fair reliability was found for gain using chin placement for all ages and in both directions. **Head impulse velocity and reliability:** Poor reliability was found for head impulse velocity for all ages and using both hand placements for rightward impulses. For leftward impulses, poor reliability of head impulse velocity was found for the younger age group with both hand placement techniques, but reliability was fair to good for older age groups for both hand placements. **Head impulse velocity:** Velocity significantly higher for chin vs. head placement, and for leftward impulses vs. right. Velocity lower for older age group vs. younger **Impulse direction:** Higher VOR gains for right lateral canals than left (statistical significance not reported)
Goggle slippage	Suh et al. (26)	**Instantaneous VOR gain calculation at 40, 60, and 80 ms:** For the very tight goggle condition, VOR gains were ~1,0 for all 3 time points. However, for the loose condition, VOR gains were significantly lower at 40 ms than the other two conditions, and higher than the other two conditions at 80 ms. **Goggle slippage-induced artifacts:** Most common artifacts detected were backward eye movement in direction of head movement, acceleration bumps, high gain, and deceleration bumps.
Target distance/ size	Castro et al. (27)	**Experiment 2, vHIT in darkness with patient “imagining targets at different distances”:** Same finding as experiment 1, VOR gain increased significantly as target distance decreased. Significant differences in VOR gain found in light vs. dark conditions for 20 and 40 cm distances, but not for 150. **Head impulse direction:** No significant differences in either experiment
	Jay et al. (28)	**Gender:** No significant effects of gender on VOR gain. **Age:** No significant correlations between VOR gain and age. **Head impulse velocity:** Significantly faster velocities for leftward impulses; peak head impulse velocity decreased with increases in age; peak velocity significantly negatively correlated with VOR gain for rightwards but not leftwards impulses. **Head impulse direction:** VOR gain significantly higher for right lateral than left
	Judge et al. (29)	**Visual acuity:** No significant effects of visual acuity on saccade frequency or amplitude, or VOR gain. **Controls vs. participants with vestibular loss (VL):** Those with VL had significantly higher frequency and amplitudes of saccades, and lower VOR gains in analyses of both target distance and size.
Head/eye position	McGarvie et al. (31)	**Head impulse direction:** VOR gains significantly higher for right vertical canals than left at all gaze angles, but smaller left-right difference for 40 degree gaze angle.
	Maxwell et al. (30)	**Regression slope gain:** No significant differences between the two head positions for either examiner
	Patterson et al. (32)	**Device:** Significantly higher VOR gains with EyeSeeCam than with ICS Impulse **Presence of saccades:** No reset saccades noted at any gaze angle for healthy subject group **Controls vs. participants with bilateral vestibular loss (BVL):** Those with BVL had significantly increased presence of saccades and significantly decreased VOR gain vs. healthy controls, at all gaze angles except +45°
	Seo et al. (33)	**Head velocity:** no significant differences in head impulse velocity between the two head positions **Head impulse direction:** VOR gains significantly higher for right lateral than left in both head positions
Thrust direction	Park et al. (34)	**Head impulse direction:** VOR gains significantly higher for right lateral than for left with both outward and inward impulses
	ElSherif (35)	**Head impulse direction:** No significant differences in VOR gain for rightward vs. leftward head impulses
	Nyström et al. (36)	**Peak velocity of head impulse:** No significant differences when using outward vs. inward head thrusts **Amplitude of head movement:** No significant differences when using outward vs. inward head thrusts; no significant correlation between head movement amplitude and VOR gain
Camera placement	Strupp et al. (37)	**Use of a weight to reduce camera asymmetry:** No significant differences in rightward or leftward VOR gain when using a weight vs. without weight
Predictability of thrust	Yilmaz et al. (38)	**Head impulse direction:** VOR gains significantly higher for right lateral vs. left
**Equipment differences (*****n** =* **3)**		
Software	Lee et al. (39)	No additional factors mentioned
Calculation	Janky et al. (40)	**Age:** No significant relationships between age and VOR gain, except when using 40 ms instantaneous gain calculation **Camera placement:** VOR gain significantly larger with impulses ipsilateral to side of recording
	Jacobsen et al. (41)	**Intra-examiner reliability:** No significant differences in VOR gain between different examiners for instantaneous gain calculation; experienced examiner had most reproducible results with regression gain calculation method **Head impulse direction:** VOR gains significantly higher for right lateral than left

#### 3.1.1. Study design and methodological quality

Since the primary aim of this study was to examine potential variation in VOR gain specifically for individuals without vestibulopathy, all studies included in this review had a participant group with no significant vestibular/dizzy history. Although it was not an exclusionary criterion to include an experimental group to be compared to a healthy control group, only six articles utilized a case-control methodology (8, 21, 29, 32, 33, 40). The remaining 26 articles utilized only healthy participants with no history of vestibular or balance disorder, although several articles grouped the healthy participants according to the variable of interest (i.e., age) (Table 1).

Additionally, blinding of participants or investigators was only reported in one study (22), and blinding occurred in the data analysis as it related to saccadic eye movements and artifact (not VOR gain calculation). It should be noted that blinding was not possible for the majority of studies due to the nature of the variables being studied (e.g., left vs. right head impulses, hand placement), and was often not needed in the analysis due to the fact that VOR gain calculations were completed by the software, not manually.

#### 3.1.2. Participant characteristics

Demographic information of study participants is reported in Table 1. Sample size of eligible studies ranged from 8 to 835 participants. Participants ranged in age from 5 to 96 years of age, although it should be noted that one study did not report participant age and several others reported a mean age, rather than a range (Table 1). While all studies reported the use of “healthy” participants with no current vestibulopathy, it should be noted that most studies relied on patient report for this information. Only a handful of studies confirmed this through the use of objective test measures, such as a neuro-otological exam (85), examination for spontaneous nystagmus (74,230), cVEMP, and/or calorics (212). One study reported confirming normal VOR gain function with vHIT as a pre-experimental measure (2,215). Additionally, one study asked participants to complete the Dizziness Handicap Inventory (DHI), excluding those with a score >14 (indicating dizziness handicap) (320). Of the studies relying solely on patient report, the inclusionary criteria varied somewhat between groups with some excluding only vestibular diagnoses, and others including measures of hearing, balance, gait, central disorder, and/or visual acuity.

#### 3.1.3. Protocol differences

While vHIT test procedure is largely normalized, there were some minor variations in protocol among studies (Table 1), including: differences in distance from visual target (ranging from 0.1 to 3.1m), number of impulses per canal (ranging from 6 to >20), the number of examiners (ranging from 1 to 4), head movement velocity (ranging from 50 to 300 deg/s), and canal tested. In regard to head impulse velocity, only two studies (7, 27) included head impulses below 100 deg/s, a range in which VOR gain could be influenced by the smooth pursuit system. Although it is typically recommended that the vHIT be assessed with higher velocity head impulses, these two studies also included trials with head impulse velocities >100 deg/s, and therefore were not excluded from this review. It should be noted that, in some articles, some of these protocol details were not reported (Table 1). Additionally, in some studies, some of these parameters acted as an independent variable that was manipulated (Table 2). A summary of the relevant secondary findings is provided in Table 2.

### 3.2. Primary factors affecting VOR gain

Overall, a total of ten primary characteristics emerged that showed a significant effect on VOR gain in a healthy sample. Primary factors that were found to contribute significantly to overall increased VOR gains were: increased patient anxiety/arousal levels, head-hand placement (for lateral impulses), decreased target distance, outward (vs. inward) thrusts for lateral canals, camera placement on adducting eye, use of position gain calculation (vs. instantaneous or area under curve calculation), and gaze alignment in the canals of stimulation. Factors contributing to overall decreased VOR gains were increasing age (over 60 to 70 years), chin-hand placement (for lateral impulses), increased target distance, gaze not aligned with canal being tested, inward thrusts (for lateral canals), camera placement on abducting eye, and gain calculation using instantaneous (especially 40 ms) or area under curve calculation. Other factors found to play a role in VOR gain differences obtained were use of multiple examiners, or use of a single inexperienced examiner, and goggle tightness. Differences between examiners or within a single examiner were more pronounced in vertical canal assessment compared to lateral canals. Additionally, very tight goggles were recommended to obtain the most accurate results. No significant differences in VOR gains were noted when comparing visual acuity (use of contact lenses vs. glasses vs. controls) (22), test-retest reliability (gains were stable across multiple sessions) (20, 21), target size (28, 29), or predictability (e.g., no significant gain difference when patients had foreknowledge of timing and direction) (38). See Table 3 for a summary of relevant findings.

**Table 3:** Factors identified through systematic search and relevant findings.

**Factor affecting VOR gain**	**Reference**	**Relevant findings**
^*^Age (*n* = 8)	Abakay et al. (14)	No significant differences in VOR gain across age groups (12–88)
	Kim and Kim (15)	^*^Patients over 70 showed a significant decrease in VOR gain in the horizontal canals. For vertical canals, gain value was relatively maintained until 80, but then significantly decreased.
	McGarvie et al. (6)	No significant differences across age groups into the 80s in the horizontal and anterior canals, and only weakly significant for posterior canal
	Matiño-Soler et al. (7)	No significant differences in gain for any head velocity until age 70; after age 70, gains begin to decrease for higher velocity head movements, but remain stable until age 90 for lower velocity head movements
	Mossman et al. (16)	Statistically, but not clinically significant gain decreases at 80 ms and 60 ms with increasing age (up to age 60)
	Pogson et al. (17)	Increased gain symmetry (due to decreased gain in left posterior canal and increased gain in left anterior canal) with increasing age over 60
	Treviño-Gonzalez et al. (18)	Slight, but significant decrease in VOR gain with increasing age, but may not be clinically significant until 79+
	Yang et al. (19)	No significant differences in VOR gain across age groups (20–69)
***Conclusion:** Gains appear to remain stable until at least the age of 60, and even then, decreases in gain are small*. ***Recommendation:** Each clinic should establish normative data for different age ranges*.		
Test/Retest reliability	Bansal and Sinha (20)	No significant gain differences for any canal between first and second test sessions
	Singh et al. (21)	Excellent test-retest reliability for all canals across four different sessions.
***Conclusion:** Test-retest reliability is good across multiple sessions for all canals*		
Visual acuity	van Dooren et al. (22)	No significant differences between individuals with normal vision, individuals wearing spectacles, and individuals wearing contacts.
***Conclusion:** No correction is needed for vHIT when testing subjects using corrective lenses*		
^*^Mental state/Anxiety	Naranjo et al. (23)	Significantly increased VOR gains noted with increased postural threat, with significant correlations between changes in electrodermal activation (measure of autonomic response) and VOR gain (for vertical canals)
***Conclusion:** Heightened state of arousal/anxiety leads to increased VOR gains* ***Recommendation:** Steps should be taken to mediate heightened levels of anxiety, including thorough explanation or test protocols and procedures and use of practice trials*		
^*^ Examiner reliability	Abrahamsen et al. (25)	Good intra- and inter-examiner reliability for horizontal canals for both systems assessed (ICS Impulse and EyeSeeCam). For vertical canals, intra- and inter-examiner reliability remained good for the ICS Impulse but showed much more variability when assessed with the EyeSeeCam. Less-experienced examiners showed more variability than more experienced examiners.
	Mutlu et al. (24)	Significant differences noted between examiners for both lateral canals, right anterior, and left posterior
***Conclusion:** Inter-examiner reliability is poorer for vertical canals compared to lateral canals, although there are some differences in outcome across different equipment, especially for vertical canals*. *Intra-examiner reliability is poorer with less-experienced examiners*. ***Recommendation:** The same examiner should be used in comparisons across individuals and between assessments, if possible*		
^*^Hand placement	Fu et al. (8)	Higher gains for head placement compared to chin/jaw placement for lateral canals
	Patterson et al. (9)	Higher gains for head placement compared to chin/jaw placement for lateral canals
***Conclusion:** Hand-head placement leads to higher VOR gains compared to chin/jaw placement for lateral canal stimulation* ***Recommendation:** Examiners should use consistent hand placement during evaluations*		
^*^Goggle Tightness	Suh et al. (26)	Very tight goggle straps lead to most accurate gains in relation to head movement at all three time points measured
***Conclusion:*** *Goggle strap tightness affects VOR gain, with very tight goggles leading to most accurate gains in relation to head movement* ***Recommendation:*** *Ensure very tight goggle fit, possibly by using a pressure gauge to ensure adequate tightness*		
^*^Target distance/size	Castro et al. (27)	Lateral canal VOR gain increased significantly as target distance decreased
	Jay et al. (28)	No significant differences in VOR gain for different target sizes
	Judge et al. (29)	Lateral canal VOR gain increased significantly as target distance decreased, with gains closed to 1 at medium target distances (1.2 m)
***Conclusion:** VOR gains for lateral canals are significantly affected by target distance, with gains closest to 1 at distances of 1–1.5 m* ***Recommendation:** Target distance should be consistent, with placement distance of 1–1.5 m*		
^*^Head/Eye Position	McGarvie et al. (31)	Vertical canal gain decreased as horizontal gaze angle shifted away from alignment with the canal plane tested
	Maxwell et al. (30)	No significant differences in lateral canal gains when tested at earth horizontal vs. 30 degree flexion positions
	Patterson et al. (32)	Stepwise gain reduction noted in vertical canals as gaze moved away from the plane of canal stimulation
	Seo et al. (33)	Wider gain value distribution in healthy individuals for lateral canals in a “head up” (0 deg) position compared to a “head down” (30 degree flexion) position. In patients with vestibulopathy, lower gains were noted on the affected side in the head down position
***Conclusion:** Gaze direction aligned with the canal plane being tested results in higher VOR gain values for both lateral and vertical canals* ***Recommendation:** For optimal gain, align gaze direction with the canal plane being assessed*		
^*^Thrust/Impulse direction	ElSherif (35)	No significant differences in gain between outward vs. inward head thrusts for left or right lateral canals
	Nyström et al. (36)	Outward head thrusts were slightly but significantly larger than inward thrusts bilaterally for lateral canal stimulation, but only for right-sided thrusts (with camera placed on left eye)
	Park et al. (34)	Outward head thrusts were significantly larger than inward thrusts bilaterally for lateral canal stimulation
***Conclusion:** Outward head thrusts show higher gains for lateral canal impulses compared to inward thrusts* ***Recommendation:** Use outward (starting at midline) thrusts, although since the clinical difference is generally small, inward thrusts may be used for patients with cervical issues*		
^*^Camera placement	Strupp et al. (37)	Higher gains noted for head impulses toward recorded eye (leftward impulses show higher gains when camera is on left eye, rightward impulses show higher gains when camera is on right eye)
***Conclusion:** Lateral gains are higher for impulses toward the same side as the recorded eye* ***Recommendation:** Use consistent camera placement*		
Predictability of impulses	Yilmaz et al. (38)	No significant effect of foreknowledge of timing or direction
***Conclusion:** Foreknowledge of impulses does not significantly affect VOR gain* ***Recommendation:** Since no differences were noted, it may be better for patients with anxiety or cervical issues to be given a forewarning for each impulse* ***Caution:** Other studies in patients with UVL have shown small but significant increases in VOR gain when impulses are predictable toward the ipsilesional side (42)*		
^*^Software/Calculation method	Jacobsen et al. (41)	Regression gain calculation was found to be more reproducible than instantaneous gain; for instantaneous gain, 40 ms was found to be significantly less reproducible than 60 or 80 ms
	Janky et al. (40)	Position gain calculation showed highest gain, followed by instantaneous gain at 80 ms, followed by area under curve calculation
	Lee et al. (39)	Significant gain differences were found within one device depending on calculation method, and gain differences were found between different equipment using the same calculation method (area under curve)
***Conclusion:** Different systems calculate gain differently* *Different calculation methods yield different VOR gains* ***Recommendation:** Since different equipment uses different software and calculation methods, it is recommended that each clinic obtain normative data for each individual device*		

Factors with significant findings are marked with an asterisk (^*^).

### 3.3. Secondary factors affecting VOR gain

As stated above, many studies included in this review examined the effects of multiple variables on VOR gain. While classification of articles was determined by what authors deemed to be the primary factor examined (based on title, abstract, findings, and discussion), efforts were made to address significant secondary findings in the studies as well. Through the analysis process, it was noted that multiple secondary factors were addressed, including canal stimulated (Table 2), head impulse velocity (7, 17, 28), gender (7, 14, 28), handedness of examiner (24), room lighting (27), and monocular vs. binocular recording (22). The majority of these secondary factors showed no significant difference in overall VOR gain. However, two secondary factors were revealed to have a significant effect of VOR gain: head impulse velocity and canal stimulated. Head impulse velocity was found to be negatively correlated with VOR gain (7, 17, 28), and canal of stimulation was reported in multiple studies as a significant finding (Table 2). Additionally, significant secondary factors in studies that were already addressed as primary factors in other articles are discussed below in the appropriate category.

### 3.4. Factors related to variation in saccades

Eleven of the 32 studies evaluated in this review reported the effects of specific factors on variation in saccade characteristics. Five of these articles were primarily focused on two participant characteristics, age and test-retest reliability. One article was primarily evaluating a tester characteristic, examiner reliability. The remaining five articles were focused on protocol differences, including hand placement, target size, and gaze angle. None of the articles in this review focused on equipment/calculation differences reported findings related to saccades.

## 4. Discussion

Of the fourteen primary factors (32 articles) noted in this review, ten factors emerged that could potentially significantly affect VOR gains in a normative population (Table 3). While higher VOR gains are often viewed as a better outcome, it should be noted that, in some studies, increased gains were not always viewed as the ideal, as some authors indicated that certain factors may be leading to “overhigh” or inaccurate gains. For example, Fu et al. (8) suggested that head-hand placement may be leading to overhigh gains that were actually inaccurate, and that jaw/chin-hand placement was potentially more reliable. Additionally, when examining target distance, it was consistently found that decreased target distance leads to increased VOR gains. However, Judge et al. (29) revealed that target distances just over one meter elicited VOR gains closest to 1, and Curthoys et al. (3) recommend a distance of no < 1 m to ensure the most accurate gains.

Based on this review, recommendations are made to assist in minimizing VOR gain variability during vHIT testing (Table 3). The following recommendations are the authors' suggestions of clinical protocols that could be implemented to reduce VOR gain variability in a non-pathological population, and are based on a review of the significant findings included in the relevant articles obtained through this systematic search. Of note, a repeated suggestion and, we believe, a key takeaway point, is that each clinic should establish their own normative data for each age group that will be tested and for any change in tester, protocol, or equipment.

### 4.1. Participant factors

Of the five major subcategories falling under the *Participant Characteristics* classification, only two, age and participant anxiety/arousal level, were found to lead to significant differences in VOR gain. Eight articles were found that primarily examined participant age, and it should be noted that the results were somewhat mixed. Pogson et al. (17) found increased variability in VOR gains with age >60 years for all canals except the right posterior, though symmetry between canals for vertical impulses increased with increasing age. Matiño-Soler et al. (7) reported decreased gains in patients over the age of 70, but only for high velocity head movements (and over 90 for lower velocity head movements). Kim and Kim (15) found significant decreases in VOR gain in the horizontal canals beginning after the age of 70 and in the vertical canals after the age of 80. McGarvie et al. (6) revealed that, into the 80s, age was not a significant factor for anterior or horizontal canals, and was only weakly significant for posterior canal gains. Mossman et al. (16) reported slight decreases in lateral canal gain with increasing age, but stated that with careful attention to methodology, the lower limit of “normal” (using a 2 SD limit) remains robust into the 70s. In accordance with this finding, Treviño-González et al. (18) found modest but statistically significant decreases in VOR gain (for the left lateral canal) and in median gain at 60 ms (for both left and right lateral canals) with increasing age. However, as the differences they found were slight, authors suggested that the decreases in VOR gain may not be clinically significant until over at least 79 years of age, the maximum age of subjects in their study. In contrast, other studies (14, 19) reported no statistically significant differences in VOR gains across age groups; however, it should be noted that Yang et al. (19) only examined participants into the 60s. Because the studies that showed significant differences in gains were all in patients over the age of 60 years, it is possible that studies examining patients only up to the age of 60 or just over are missing some of the age-related decreases in gain. Further, although not the primary factor investigated in the study, Jay et al. (28) found that, when controlling for head velocity, age effects in VOR gain could be seen earlier, and suggested that, similar to the findings of Matiño-Soler et al. (7), effects of age and head velocity are correlated, even potentially in individuals under the age of 70. Based on review of these studies, it appears that age related changes in VOR gain may be somewhat complex, and potentially multifactorial. It does appear that given a consistent protocol, below the age 60 years, VOR gains are stable, but even beyond this, gain decreases are small, and likely to be clinically non-significant. However, because of the mixed nature of results in prior studies, and the potential that multiple interacting factors are at play, it is the recommendation of the authors that each clinic should establish their own age-related normative data.

In examining participant anxiety/arousal level, only one study was found, which compared vestibular evaluation results obtained with participants on a low platform (0.8 m from ground) and a high platform (3.2 m from ground) to increase what authors termed the “postural threat” (23). Results showed significantly increased VOR gains for both vertical and lateral canals with increased postural threat. This finding provokes an interesting question: “Why might clinicians see an ‘increased' physiologic response (high VOR gain) in response to fast, reflexive movements that are largely independent of central modulation?” The literature on this topic seems to show somewhat mixed results, as prior literature examining vHIT and VEMP in patients with panic disorder compared to controls showed no significant differences in outcomes, although higher vHIT VOR gains correlated with higher levels of postural instability (43). Additional studies examining functional HIT (f-HIT) found that only anxiety levels prior to testing were predictive of worsening f-HIT outcomes with optokinetic stimulation present, and authors suggested that increased anxiety may play a role in visuo-vestibular interactions (44). Therefore, it does seem possible that higher levels of state anxiety may keep the system in a higher state of arousal, thereby leading to changes in VOR gain function, even at the reflexive level. However, further research is needed to elucidate these findings.

Along this same line, Yilmaz et al. (38), in examining the effect of predictability of impulses (timing and direction), found no significant effects of foreknowledge of impulses, so it is possible that a “forewarning” of impulses for patients with high levels of anxiety could be beneficial in alleviating the anxiety response. However, this recommendation must be taken with caution, as some studies in patients with UVL have shown small but significant gain increases when impulses are predictable toward the ipsilesional side (42). Other studies in patients without vestibulopathy have found non-physiological abnormally high VOR gains in the lateral canals when impulses are predictable (16). Therefore, further research is needed in this area, especially in patients with vestibulopathy. Until that time, a thorough explanation of test protocols and procedures, and use of practice trials may help alleviate anxiety in patients, leading to potentially more accurate results.

### 4.2. Tester factors

Only two articles were found to fall under the category of *tester characteristics*. Both studies examined inter-examiner reliability (24, 25), and both concluded that inter-examiner reliability is poorer when examining the vertical canals. Although one study found significant differences between examiners for both the lateral and vertical canals, higher inter-class correlation (ICC) values for the lateral canals indicated greater VOR gain variability for the vertical canals (24). Similarly, in a comparison of the EyeSeeCam (Interacoustics) and ICS Impulse (Otometrics), good intra- and inter-examiner reliability was found for the lateral canals with both devices, but only when using the Impulse for the vertical canals, indicating that greater variability may be present with the vertical canals and for the EyeSeeCam (25). Additionally, in examining intra-examiner reliability, more variability in gains was noted with less experienced examiners, leading authors to speak to the importance of experience and training in minimizing variability in results (25). It should be noted that a separate study examining equipment differences did not find any statistically significant differences between examiners, although authors did note that the experienced examiner had more reproducible results than the inexperienced examiner (41). Additionally, although not the primary purpose of the study, Patterson et al. (9) found that there was overall poor inter-examiner reliability in terms of head velocity, suggesting that different examiners tend to stimulate head movements at varying velocities. Given this information, it is recommended that the same examiner conducts vHIT testing when reliability is a concern, such as in measuring a single patient's progress across a management program, or when comparing results across individuals.

### 4.3. Protocol factors

A total of fifteen articles were reviewed primarily discussing differences in protocol that can affect vHIT. Of these, two articles examining target size found that target size has no significant effects on VOR gains (28, 29). Additionally, one article examining predictability of impulses found no significant gain differences between predictable and unpredictable impulses (38), although as mentioned previously, this finding should be interpreted with caution, as other studies have reported increased gains with predictable impulses. The remaining thirteen studies found significant differences in gain based on protocol differences such as hand placement (2), goggle tightness (1), target distance/size (2), head/eye position/alignment (4), thrust/impulse direction (3), and camera placement (1).

Both Fu et al. (8) and Patterson et al. (9) concluded that VOR gains were higher with a head-hand placement (both hands placed on top of patient's head) compared to a chin/jaw-hand placement (both hands placed along patient's jaw). However, as mentioned previously, Fu et al. (8) cautions that the head-hand placement may be leading to overhigh VOR gains due to the finding of a significant number of impulses showing gains >1. Authors attributed this finding to the possibility of goggle movement from quick head thrusts having more of an effect with head-hand placement. Indeed, in a study examining goggle slippage, Suh et al. (26) found that VOR gain when using a loose strap pressure was significantly lower than VOR gain with a tight or very tight strap pressure, with an instantaneous gain calculation at 40 ms. When instantaneous VOR gain was calculated at 80 ms, a significant negative correlation was found between VOR gain and goggle tightness, in that VOR gain decreased as goggle strap pressure increased. However, with the loose strap pressure and the 80 ms gain calculation, VOR gains were, as authors suggest, “overhigh,” with an average gain of 1.24. Therefore, as the goggle tightness increased, VOR gains came closer to the expected value of 1.0. Additionally, with loose goggle strap pressure, several artifacts appeared in the vHIT tracings, while the tracings obtained with very tight strap pressure were artifact-free. Authors attributed these findings to a possible slingshot-like motion of the goggles during head thrusts. Overall, it was recommended that the goggles be very tightly affixed to the individual's head to avoid major changes in VOR gain due to goggle slippage. Authors suggested that using a pressure gauge to ensure efficient goggle tightness may reduce the likelihood of slippage, and recommend a pressure level of at least 45 cm H20 to produce very tight strap pressure. Curthoys et al. (3) state that a “telltale sign” of goggle slippage occurs when the eye velocity recording begins before the head velocity recording onset, and strongly recommend ensuring a very tight goggle fit prior to initiating testing. Additionally, although head-hand placement is often recommended, care should be taken in use of this technique for specific patients, and once again, each clinic should establish norms for the desired hand placement. Because factors such as poor fit and/or slippage of goggles, loose skin, and hair texture can introduce artifact into the recording, it is also important for the clinician to manually examine tracing curves provided by the software, rather than relying solely on calculated gain values (3, 45).

For target distance, both Castro et al. (27) and Judge et al. (29) found statistically significant increases in VOR gains as visual target distance decreased. Judge et al. (29) reported gains closest to 1 (mean = 0.98) in a healthy control group when the target distance was at a medium distance from the patient (1.2 m). Curthoys et al. (3) also recommends target distances of no closer than 1 m, and most studies and clinical recommendations utilize a target distance of 1–1.2 m (45).

Different equipment manufacturers offer different guidelines for head/eye position. For example, the EyeSeeCam (Interacoustics) recommends an initial head position of 0 degrees azimuth (center gaze) for vertical canal stimulation, while the ICS Impulse (Otometrics) recommends an initial head position of 45 degrees relative to the target. Additionally, there has been some question regarding lateral canal stimulation with regard to the use of an earth horizontal head position vs. a 30-degree head flexion (to place the lateral canals horizontal to the ground). For vertical canal stimulation, both McGarvie et al. (31) and Patterson et al. (32) found a significant stepwise reduction in gain as gaze moved away from the plane of the canal being stimulated, with ROC curve analysis suggesting performance closer to chance when gaze is opposite the canal of stimulation (32). For lateral canal stimulation, studies show somewhat mixed results, with Maxwell et al. (30) showing that no significant differences in VOR gain were present between earth horizontal and 30-degree flexion positions. However, Seo et al. (33) revealed that a 30-degree flexion of the head (head-down position) produced more reliable gain values with smaller standard deviations compared to a 0-degree flexion (earth horizontal) position. Based on these findings, it is recommended that for optimal gain, eye gaze direction should be aligned with the canal plane being stimulated for both vertical and lateral canals.

Studies on thrust/impulse direction show somewhat mixed results with Park et al. (34) showing significantly higher VOR gains for outward thrusts (from midline to lateral position) compared to inward thrusts, and ElSherif (35) reporting no significant differences in VOR gains for thrust direction. Nyström et al. (36) also showed a statistically significant larger VOR gain for outward impulses, but indicated that the difference was so small that both inward and outward thrusts should be acceptable for clinical use. Several factors were purported to affect these differences, including increased neck tension and increased physiologic startle, which could lead to increases in VOR gain. Importantly, although the focus of this review is on patients without vestibulopathy, Nyström et al. (36) noted the possibility of Alexander's Law playing a role in patients with peripheral vestibulopathy, as well as the possibility of cervicogenic disorder contributing to asymmetries on vHIT testing when the impulses end in a lateral gaze position. Therefore, the authors recommended that a neutral-gaze ending position (as seen in inward thrusts) may be less affected by non-vestibular factors, and suggested the use of inward thrusts for patients with a history of neck pain or trauma. It is our opinion that outward impulses tend to show more reliable and slightly increased VOR gain values compared to inward impulses. However, we recognize that caution should be taken in interpreting results in patients with spontaneous or gaze-evoked nystagmus, and in patients with cervicogenic disorders, in which case inward impulses may be a plausible alternative.

Multiple studies have found significant differences in lateral canal VOR gains between left- and right-sided impulses (Table 2), with most studies concluding that right-sided impulses show statistically higher VOR gains than left-sided impulses. Strupp et al. (37) aimed to address this finding by examining gains from two systems, the ICS Impulse (Otometrics), which has a fixed camera placement over the right eye, and the EyeSeeCam (Interacoustics), which has a moveable camera. Findings revealed that when the camera was placed over the right eye (as it often is in many systems), rightward impulses showed higher VOR gains compared to leftward gains. However, when the camera was placed over the left eye, leftward impulses showed higher gains. As Strupp et al. (37) postulated, it is likely that gains are higher for the adducting eye due to increased latencies. This finding confirmed prior findings by Janky et al. (40), who found that gains were larger with impulses in the same direction as the measured eye. The finding that measured gains are larger in the adducting eye helps explain the gain differences noted between different canals in lateral impulses. Additionally, McGarvie et al. (31) proposed that differences in vertical canal gains could be explained by differences in eye rotation increases from recording only one eye. These findings suggest that camera placement over the left or right eye is likely inconsequential as long as camera placement is consistent. Additionally, van Dooren et al. (22) found no significant differences between monocular and binocular recordings, suggesting that even in monocular recordings over one eye, the differences are minimal. Although most seem to agree that gain differences due to camera placement are clinically non-significant, it is important to note that, although not included in this review, remote camera systems have been found to potentially alleviate the gain asymmetry issue, since these systems always record the eye ipsilateral to the canal being tested (12).

### 4.4. Equipment factors

Three studies were found examining differences in equipment, including differences in both software (39, 40) and VOR gain calculation method (39, 41). Lee et al. (39) compared two different devices and software programs, the ICS Impulse (Otometrics) and the SLVNG vHIT (SLMED, Inc). Results showed higher gains from the ICS Impulse compared to the SLVNG system when using the same gain calculation method (area under curve-AUC). Additionally, within one system (SLVNG), higher overall gains but smaller standard deviations were found using the AUC gain calculation method compared to the instantaneous peak velocity calculation. Janky et al. (40) compared gains using three different calculation methods: AUC (ICS Impulse), instantaneous velocity at 80, 60, and 40 ms (EyeSeeCam), and position gain (Visual Eyes, Micromedical). Results showed that use of position gain calculation showed significantly higher VOR gains than the other two methods, and that instantaneous velocity at 80 ms showed significantly higher gains compared to AUC calculation. Authors did caution, however, that multiple factors could have contributed to these outcomes, given the additional differences between devices, and further research is needed to expound on these findings. In comparing regression gain to instantaneous gain at 80, 60, and 40 ms, Jacobsen et al. (41) found that regression gain was found to be more reproducible than instantaneous gain, and that instantaneous gain at 80 and 60 ms was more reproducible than instantaneous gain at 40 ms. While examining age effects on VOR gain, Pogson et al. (17) also found significant differences in calculation method, with a method using a wider calculation window finding higher gains than methods using earlier and more narrow windows. Curthoys et al. (3) recommend the use of an area gain measure, in which the whole de-saccaded eye movement is divided by the whole head movement, in order to eliminate potential artifact inherent in momentary or instantaneous measurements. However, some studies have indicated that the use of earlier instantaneous measurements may be a better indicator of endolymphatic hydrops (46), suggesting that some gain calculation methods may be better for diagnosis of certain types of vestibulopathy. Additionally, while it has been established that vHIT testing shows good agreement with scleral search coil systems (13), to our knowledge, this finding has not been replicated with different systems. It should further be noted that Patterson et al. (32), in comparing the ICS Impulse and EyeSeeCam systems, found higher gain values, but also but lower intra-rater reliability for the EyeSeeCam; however, the authors state that both devices worked to separate normal from abnormal responses. Similarly, in a comparison of these two devices, poorer intra- and inter-examiner reliability was found for the vertical canals using the EyeSeeCam than with the ICS Impulse, though reliability was good for the lateral canals with both devices (25). It is also of note that there is a third vHIT device called the SYNAPSYS, which, to our knowledge, has not yet been used in comparison to other vHIT softwares in a normal sample. Articles using the SYNAPSYS system only were excluded from this review, since VOR gain is calculated using an external camera, different from other devices with a head-mounted camera. However, in one study of subjects with bilateral vestibulopathy, vHIT results were compared in three devices: the ICS Impulse, the EyeSeeCam, and the SYNAPSYS. Results of this study showed that while calculated VOR gain was similar between the Impulse and the EyeSeeCam, VOR gain obtained by the SYNAPSYS device was statistically significantly decreased (47). A comparison of the three devices in an asymptomatic sample would be needed to determine if gain is also significantly different for the SYNAPSYS system in those without vestibulopathy. Further research is still needed examining calculation methods and equipment software. Until further data establishes clear advantages of one method over another, it is recommended that each clinic establish normative data for each individual device, and that caution be taken in comparing gains between devices.

### 4.5. Secondary factors

Of multiple secondary factors identified through the review process, only two revealed significant findings suggesting a possible significant effect on VOR gain: head impulse velocity and canal of stimulation. Matiño-Soler et al. (7) compared the effects of four different head impulse velocity ranges (70–90, 100–120, 140–160, and 180–200 deg/s) on VOR gain, and reported that VOR gain decreased as impulse velocity increased across all ages and regardless of sex. It should be noted, though, that these decreases in VOR gain actually brought the values closer to 1.0, as gains were higher than 1.0 with lower head velocities, nearing 1.20 with the 70–90 head velocity range. Pogson et al. (17) also found a negative relationship between head velocity and VOR gain, as did Jay et al. (28), though only for rightward impulses. Due to the possibility of obtaining overhigh VOR gains with lower head velocities, it is recommended that clinicians attempt to produce higher head impulse velocities when possible. Higher head velocities (~150–200 deg/s) are also recommended for clinical practice by Curthoys et al. (3), due to the possibility of missing a unilateral vestibular lesion with lower velocities. Producing higher velocity head impulses can also limit the potential of influence on VOR gain from the smooth pursuit system, which could be activated with impulse velocities < 100 deg/s. In fact, past research has shown that the smooth pursuit system can produce gains as high as 0.9 for target velocities at least up to 75 deg/s (48), and potentially even up to 90 or 100 deg/s in some individuals (49).

As noted previously, two of the articles evaluating head impulse velocity as a secondary factor also found a correlation between head impulse velocity and age, where head impulse velocity decreased with increasing age (7, 28). Both studies suggested that higher velocity impulses may be more difficult to deliver in older adults who may have age-related cervicogenic deficits, therefore leading to higher gains. In accordance with these findings, Patterson et al. (9) found that for both hand placement techniques evaluated, head impulse velocities were significantly lower for the older subject group than for younger subjects, but VOR gains were higher for older subjects. Jay et al. (28) proposed that the inability to produce high-velocity head impulses in older adults may somewhat “mask” the true effects of age-related declines in vHIT VOR gain. Indeed, when head impulse velocity was controlled in this study, small but statistically significant age-related decreases in VOR gain were noted in patients >58 years of age. Similarly, Matiño-Soler et al. (7) reported a significant decrease in VOR gain for subjects >71, but only for the highest head velocity range (180–200 deg/s). With the two lowest head velocity ranges (70–90 and 100–120 deg/s), VOR gain did not decrease significantly until age 90. The above findings suggest that, in clinical practice, it is ideal to produce higher-velocity head impulses, but to be aware that gains could be inflated in older patients where producing such movements may not be possible. It should further be noted that, although here head velocity is discussed as a causative factor, it was more often utilized as a dependent variable (8, 33). See Table 1 for reported velocity differences among studies.

As discussed previously, differences in VOR gain resulting from different canal of stimulation (Table 2) are largely explained by differing gains in the adducting vs. abducting eye, leading to greater gains for the eye on the same side as the impulse (e.g., right eye shows greater gains on rightward impulses). If the camera is only recording from one eye, findings would suggest higher gains for the ipsilateral side of stimulation.

### 4.6. Factors related to variation in saccades

While examination of refixation saccades was not the primary focus of this review, we recognize the clinical importance of including this metric. Therefore, we have included a brief, but not comprehensive, review of saccadic examination in the included articles. Articles examining saccades as a secondary factor fell into three of our major categories related to variation in vHIT: participant characteristics, tester characteristics, and protocol factors.

#### 4.6.1. Participant characteristics and saccades

In four studies evaluating the effects of age on vHIT results, corrective saccades were evaluated in addition to VOR gain (7, 17, 19, 24). Two of these studies found that saccades were impacted by age, with Matiño-Soler et al. (7) reporting increased presence of saccades in participants over the age of 71 compared to those under 71, and in those over the age of 41 compared to those under the age of 41. In agreement with this finding, Pogson et al. (17) reported that saccades increased in frequency, amplitude, and peak velocity for older subjects. However, Yang et al. (19) found no significant differences in saccade presence or amplitude based on age in their study of participants from age 20–69. In this study, saccades were present in ~23% of head impulses overall, with similar saccade frequency across all age groups. Mossman et al. (16) did not specifically report the effects of age on saccades, but suggested that use of an instantaneous gain calculation at 60 ms may be best for vHIT analyses when saccades are present, due to vHIT calculated at 80 ms being more greatly affected by presence of saccades. In Pogson et al. vHIT analysis using the 60 ms gain calculation, it was found that saccade amplitude and peak velocity were strongly related to VOR gain, and saccade frequency and onset latency were moderately related to VOR gain, validating the use of this gain calculation method for diagnosing vestibulopathy with vHIT. Pogson also noted that saccade frequency increased with increases in head impulse velocity, with the strongest effect of this result in the lateral canals across the age range. Additionally, the largest saccades were found in the lateral canals, followed by the posterior canals, then anterior canals. Also, the effect where saccade frequency increased with increasing age was strongest for the lateral canals (17).

In an analysis of test-retest reliability, Singh et al. found no refixation saccades in any of their 20 healthy subjects. For individuals with vestibulopathy however, saccades were present in 46–57% of affected ears, and in 75% of unaffected ears, across four test sessions. Saccades were most commonly found, and were most consistent across trials, in lateral head impulses. Authors suggested that refixation saccades are most reliable and repeatable with lateral canal vHIT, showing moderate to excellent reliability; however, vertical canal vHIT should be interpreted with caution in the presence of catch-up saccades used to diagnose vestibulopathy, which here showed poor to moderate reliability in vertical canals (21). These findings are consistent with those of Pogson et al. (17) which found that saccade effects were strongest for the lateral canals.

#### 4.6.2. Tester characteristics and saccades

In Abrahamsen's analysis of examiner reliability (25), only 12 of their 210 participants had saccades, and only three of these had reduced VOR gain along with saccades. Only two showed saccades in the same canal tested across all four examiners. The authors here suggested that these saccades found in these healthy individuals were likely artifact resembling saccades, due to these individuals' lack of reported vestibular symptoms. In this study, greater variability was found overall in the vertical canals compared to the lateral canals, possibly due to the difficulty of delivering consistently high-velocity head impulses in vertical canal vHIT. The authors suggested that delivering high-velocity head impulses in vertical canals is important for detecting presence of saccades, and that pathological saccades could be missed if head impulse velocity is too low, again validating that vertical canal vHIT should be interpreted with caution in diagnoses of vestibulopathy (25).

#### 4.6.3. Protocol differences and saccades

Fu et al. compared two hand placement methods, jaw hand placement and head hand placement, in control subjects and those with unilateral vestibular neuritis (UVN). No saccades were detected in any control subjects. For subjects with UVN when using head hand placement, 21 of 67 individuals had saccades, but all also had normal VOR gain values. When using jaw head placement, 11 of those same subjects had saccades, but still had normal VOR gain values. Typically in vHIT assessments, vestibulopathy is diagnosed when both saccades are present and VOR gain is low; however, the authors here speculated that normal VOR gain values may be found in the presence of corrective saccades in some periods of recovery from a vestibular lesion (8).

Judge et al. evaluated the effects of target size on vHIT in a group of controls vs. those with vestibular lesions, and found that those with vestibulopathy had statistically significantly increased saccade amplitude and frequency compared to controls. However, they found no significant effects of target size on saccade frequency or amplitude (29). Jay et al. (28) also evaluated effects of target size, as well as target distance on vHIT in a group of normal subjects. In this study, all subjects had at least one saccade in at least one trial, which is a higher incidence of saccades than healthy individuals in other studies where incidence was around 25% (7, 19). In this study by Jay et al. target size did have a significant effect on saccades, where saccade incidence significantly decreased as target size increased. In their analysis using the standard target size only, this study also found that age and gender were related to saccades, with age showing a positive correlation with saccade incidence and peak velocity. Also, males had higher saccade incidence, higher saccade peak velocity, and earlier latency. However, when age was controlled in this analysis, only saccade incidence and velocity were significantly related to male gender (28).

McGarvie et al. (31) evaluated the effects of gaze angle on vHIT, and found that when gaze angle was aligned opposite the plane of the canal being stimulated, low VOR gain values were found, even below 0.5, but corrective saccades were not present. Authors suggested that this gaze angle is not valid in the assessment of vHIT, a finding validated in Patterson et al.'s evaluation of gaze angle. In Patterson's study, although no saccades were noted for any gaze angle in the healthy subject group, all subjects with bilateral vestibular lesions (BVL) showed reset saccades in the−45- and 0-degree gaze angles. In both of these gaze angles, the saccades were found in the presence of reduced VOR gain, which would be valid for diagnosis of vestibulopathy in clinical scenarios. However, no subjects with BVL had repeatable saccades with the +45-degree gaze angle (gaze opposite of canal being stimulated), though low VOR gain was often observed at this gaze angle (32). This study therefore confirms that this gaze angle is not valid for vHIT, but that either 0- or−45-degree angles can be used clinically, as both of these showed high sensitivity and specificity in Patterson's study. It was suggested in both studies that gaze angle aligned opposite of the canal being tested is not optimal for vHIT, as low VOR gain may be obtained without presence of corrective saccades that would indicate vestibular dysfunction. This low gain is thought to be associated with a large torsional eye movement that is required to maintain target fixation with this angle; although the eye movement may actually be similar in velocity to the head movement (i.e., normal gain), torsional eye movements cannot be detected by VOG techniques and low VOR gain would be measured without a corrective saccade (31, 32).

As the presence of saccades is typically considered to be an indicator of vestibular dysfunction in vHIT testing, it is important to keep these findings in mind. For instance, saccades in vertical canal vHIT should be interpreted with caution due to poorer reliability compared to saccades found in horizontal canal testing (25). Additionally, it is likely that increased saccades occur with increased age even in normal subjects (7, 17), although one study did not find differences in saccade presence due to age (19). In pathological subjects, Fu et al. (8) noted that corrective saccades may be present even with normal VOR gain. Although there is still some variation in the literature about the appearance of saccades in vHIT, consistency across patients in regard to test protocol should help to minimize variations in results.

### 4.7. Limitations

This systematic review had some limitations, including that only four academic databases were used for a search of the literature, lending the possibility that some relevant articles could have been missed. Also, the full-text review was limited to only articles that could be obtained in English. Additionally, included studies largely only evaluated a single group of healthy or normal individuals, with only six case-control studies included. Within these populations, there was significant variation on how “normal” or “healthy” was defined, with only a handful of studies confirming normal vestibular function through an objective metric. However, even among studies relying solely on patient report, there was variation in the exclusionary criteria, with some examining only vestibular disorder, and others examining multiple other factors, such as balance disturbance, central disorder, gait disturbances, visual acuity, and hearing status. Because vestibulopathy may present asymptomatically, this could be a confounding influence in the findings. Also, as this review only focused specifically on variation in VOR gain in normal individuals, it is unknown how these various factors may affect VOR gain in individuals with vestibulopathy. Additionally, although the effects of specific factors in these studies on saccades were reported, only VOR gain was used as a search term for this review, and saccade results were therefore only available for the papers in this study primarily focused on VOR gain. Future researchers may consider a similar review primarily focused on saccade characteristics. Future reviews should consider evaluating variation in VOR gain in other populations, including populations with vestibulopathies and pediatric populations, and examining additional outcome metrics, such as the presence of refixation saccades.

## 5. Conclusion

The studies included in this review examine variations in VOR gain due to participant, tester, protocol, and equipment differences in individuals without vestibulopathy. While some of the factors studied in this review are unable to be controlled in a clinical test environment, it is important to maintain a consistent and controlled test environment, so that variations in gain are minimized as much as possible. As a general rule, manufacturer protocol recommendations should be followed and each clinic should establish norms within each test facility using the same equipment, calculation method, and tester(s), whenever possible. All testers should be well-trained to minimize variations between clinicians, and should always examine the tracings provided through the software, rather than solely relying on the gain calculation provided. In summary, although some degree of variation is likely to be inevitable, studies suggest that training and consistency are key factors to obtain the most accurate and repeatable results possible.

## Data availability statement

The original contributions presented in the study are included in the article/supplementary material, further inquiries can be directed to the corresponding author.

## Author contributions

Conceptualization, methodology, validation, data curation, writing—original draft, writing—editing and reviewing, and visualization: LM-N and AF. Investigation: LM-N. Supervision and project administration: AF. Both authors contributed to the article and approved the submitted version.
